# [Corrigendum] Abnormal expression of SIRTs in psoriasis: Decreased expression of SIRT 1-5 and increased expression of SIRT 6 and 7

**DOI:** 10.3892/ijmm.2024.5472

**Published:** 2024-12-16

**Authors:** Xiaojing Fan, Kexiang Yan, Qinqin Meng, Rui Sun, Xinrong Yang, Dingfen Yulan, Fulun Li, Hui Deng

Int J Mol Med 44: 157-171, 2019; DOI: 10.3892/ijmm.2019.4173

Following the publication of the above article, the authors contacted the Editorial Office to explain that three pairs of the western blots featured in Fig. 4 on p. 165 had inadvertently been duplicated in this figure. Essentially, it appeared to the authors that the blots in Fig. 4A for SIRT5 were identical to those in Fig. 4C for SIRT6 (P2 versus C2); the blots in Fig. 4B for SIRT4 were identical to those in the same figure part for SIRT5; and the blots in Fig. 4C for SIRT6 (P3 versus C3) were identical to those in Fig. 4C for SIRT7 (P1 versus C1).

The authors were able to re-examine their original data, and identified the data that should have rightfully been included in this figure. The revised version of Fig. 4, now incorporating the correct data for SIRT6 (P2 versus C2) in Fig. 4C, SIRT5 in Fig. 4B, and SIRT7 (P1 versus C1) in Fig. 4C, is shown on the next page. The authors can confirm that the errors associated with this figure did not have a significant impact on either the results or the conclusions reported in this study, and all the authors agree with the publication of this Corrigendum. The authors are grateful to the Editor of *International Journal of Molecular Medicine* for allowing them the opportunity to publish this Corrigendum; furthermore, they apologize to the readership of the Journal for any inconvenience caused.

## Figures and Tables

**Figure 4:**
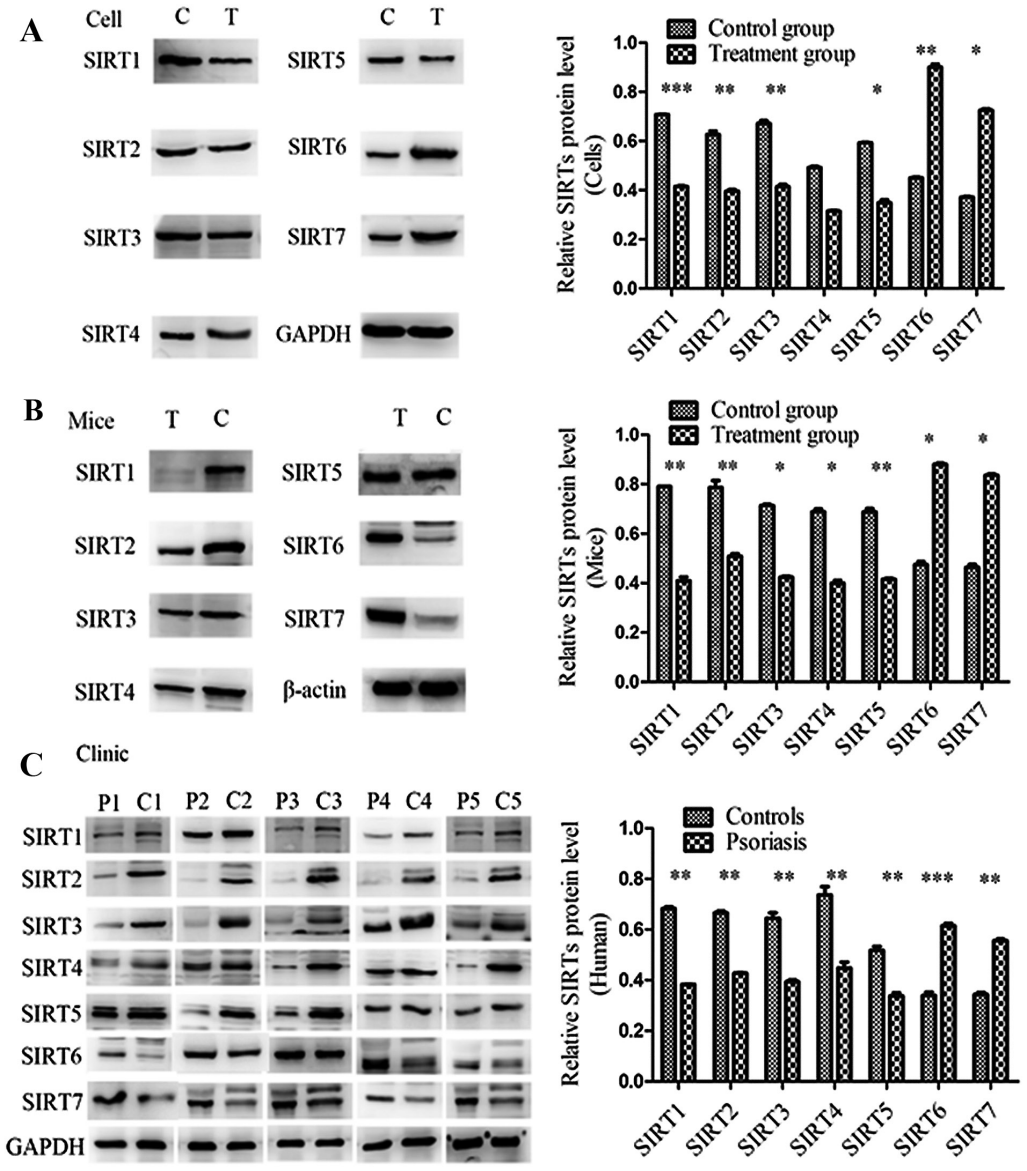
Protein expression levels of SIRTs determined by western blotting in (A) cells, (B) mouse tissue and (C) human tissue. The protein expression levels of SIRT1, SIRT2, SIRT3, SIRT4 and SIRT5 in the experimental groups/patients with psoriasis were significantly reduced and those of SIRT6 and SIRT7 were significantly increased compared with the controls. Error bars present the standard deviation. ^*^P<0.05, ^**^P<0.01 and ^***^P<0.001 vs. the control group. SIRT, Sirtuin.

